# An Account of a Case of Delirium, Produced by Belladonna and Stramonium, Presenting the Character of Somnambulism

**Published:** 1821-07

**Authors:** 


*An Account of a Case of Delirium, produced by Belladonna and Stramonium, presenting the Character of Somnambulism.* Read to the Société Medicale d'Emulation of Paris, by Dr. Sarlandiere.

On the 14th of March, 1820, at about eight o'clock in the morning, Dr. Sarlandière was called to visit a tailor, forty-five years of age, whom he found in a state of permanent convulsion, with some degree of opisthotonos; his eyes open and fixed, the pupils so much dilated that the circle of the iris was hardly visible; the limbs agitated by some "*automatic*" movements, which suddenly ceased to give place to a sort of tetanic contraction.

The wife of this man, thirty years of age, was sitting in the room; she had no convulsive motions, but her eyes were fixed, and the pupils very much dilated.

Having been informed that some glysters, which had been used a short time previously, were the cause of the state in which those persons were found, Dr. Sarlandière suspected that the nervous symptoms had been produced by a decoction of some narcotic substances, and most likely of belladonna. On examining the remains of the herbs with which the glysters had been prepared, he, assisted by Dr. Delarue, and Mr. Lalande, an apothecary, recognized amongst them *belladonna*, *datura stramonium*, and *papaver nigrum*. They immediately endeavoured to obviate the ill consequences, by the administration of purgative, and afterwards acidulated, glysters, and by acidulated drinks. The plants above enumerated had been given, by mistake, by an herbalist, who had been asked for emollients.

After the lapse of an hour, the permanently convulsive state had diminished in severity in the husband. He soon after this placed himself in his bed in the attitude he was accustomed to assume on his board, and presented a complete and perfectly characteristic state of somnambulism. He appeared to set to work at his habitual occupations, and simulated all the actions which they required. He appeared to lay out and fold pieces of cloth; handled any thing that came in the way of his fingers as if he had picked a needle out of a cushion; seemed to thread it, after having apparently drawn a piece of thread from a situation, in respect to him, where a thread-paper would have been placed had he really been on his board at work; and did as if he had made a knot at the presumed end of the thread. Sometimes, even, (imagining, no doubt, that the thread had escaped from his fingers,) he twisted it again; and it might be said, also, that he attempted to thread his needle several times, until he was assured that the thread had passed through the eye of it. It is necessary to give these minute details, in order to show how far the brain of this man was occupied by a particular series of ideas, to the exclusion of all others. All other relations with external objects was, as it were, destroyed: he did not hear, although a person hallooed into his ears: he did not see, for, whatever might be placed before his eyes, he did not regard it, and appeared to be wholly occupied by his work. Sometimes he seemed to believe that he saw some one come into the room; he saluted him, smiled, moved his lips as though he spoke to him, (for he had not the power of speech,) and listened to his answer. Sometimes he simulated the action of spitting, though he did not spit in reality; at others, he appeared to think he was taking a measure for a coat, cut with his scissors, folded his cloth, &c. and so on, without a moment's relaxation, he occupied himself in actions relative to his avocation. This state of activity continued for fifteen hours, without his appearing to desire to eat, and without his drinking any thing else than a few spoonfuls of lemonade, which he was made to swallow not without great difficulty. The somnambulism did not, however, continue in the same degree all this time, although he did not cease to work, apparently, in the same manner: he gradually recovered his voice; he then followed up a conversation, and imagined that he was replied to. It was not until the evening that he really heard what was said to him, and that he could answer in a rational manner. His sight returned somewhat later, and he then recognized very well the persons who were present. The pupils were then much less dilated. His brain was, however, pre-occupied; he continued his pretended work, and imagined he held in his hands the implements of his trade.

Dr. Sarlandière, on visiting him now, (for the sixth time this day,) found several persons about his bed, with whom he talked in a familiar manner, but still kept on at his work: they were trying, in vain, to make him understand that he had nothing in his hands, that he was abused by his imagination, and that he was not on his board, but in his bed. He only laughed at them, and said that they wanted to play their jokes with him. In this state of matters, Dr. Sarlandière thought of a means for changing the order of his ideas, that succeeded completely: he went to him, remarking that he was very hard at work that day, drew his watch from his pocket, and, having got the patient's attention fixed on it, made it strike twelve. He then blamed him for sitting so late at work, told him that it would prove injurious to his health, and that he would be fit for nothing the next day. These remarks appeared to strike him: he seemed to fold up his work, get down from his board, undress himself, and go to bed. He passed the night quietly, excepting that he was affected with dreams and some "*automatic*" movements.

On the ensuing morning his reason had returned; that is to say, he possessed completely the ordinary use of all his senses. A slight access of fever appeared; but abstinence, quietude, and acidulated drinks, promptly restored his health.

The wife suffered a degree of intellectual disturbance less violent than that of her husband. Part of her glyster had been immediately evacuated, whilst the husband had retained the whole of his. One or two minutes after the injection of it, she seemed stupefied, her arms became very heavy, it seemed to her as if they were of lead. She thought she saw at the same instant thousands of flies, to which there succeeded serpents, blazing fires, rooms hung with black; and, in the midst of these objects, she now and then perceived those which really surrounded her: she saw her room, her husband, her goods. She remained all the day sitting on a chair, believing she saw near her scissars, needles, pieces of cloth to be sown together; sometimes she endeavoured to seize these objects, that she might go to work, but they appeared to escape as she was on the point of grasping them; at other times she thought she really did take them into her hands, and then she began to make use of them. All this she afterwards told; and Dr. Sarlandière had remarked in her, actions exactly conformable with her account. The pupils were extremely dilated, her eyes fixed and wide open, as well as those of her husband; but she was more sensible of her condition, and, when she was questioned, she answered that *a glyster administered that morning was the cause of the state of* IVRESSE* *in which they found her.* Sometimes she started up, excited by an idea which occurred to her mind, and ran forward as it were terrified at something that was behind her: but she always preserved the use of her voice, her hearing, and even her sight, when her attention was strongly fixed on any object.—(*Revue Medicale*, Fevrier 1821.)

* There is no English word having exactly the same meaning as this; and we think it proper to give the original, as the woman's consciousness of her exact condition, in this respect, is a somewhat important circumstance.—Editor.

